# Absence of Genotoxic and Mutagenic Effects of *Zingiber zerumbet* (L.) Smith (Zingiberaceae) Extract

**DOI:** 10.1155/2012/406296

**Published:** 2012-07-15

**Authors:** Chia Ju Chang, Thing-Fong Tzeng, Shorong-Shii Liou, Yuan-Shiun Chang, I-Min Liu

**Affiliations:** ^1^School of Chinese Pharmaceutical Sciences and Chinese Medicine Resources, China Medical University, Taichung 40402, Taiwan; ^2^Department of Internal Medicine, Pao Chien Hospital, Pingtung County, Ping Tung City 90064, Taiwan; ^3^Department of Pharmacy and Graduate Institute of Pharmaceutical Technology, Tajen University, Yanpu Shiang, Ping Tung Shien 90701, Taiwan

## Abstract

The present study evaluated the potential genotoxicity of the ethanol extracts from the rhizome of *Zingiber zerumbet* (L.) Smith (EEZZR) using a standard battery of tests. Chemical analysis with liquid chromatography-tandem mass spectrometry revealed that EEZZR contained Zerumbone (200.3 ± 0.37 *μ*g/g) and 6-gingerol (102.5 ± 0.28 *μ*g/g). There were no increases in the number of revertant colonies with EEZZR at concentrations of 150–5000 *μ*g per plate, regardless of the metabolic activation system (S-9 mix) used in the histidine-dependent auxotrophic mutants of *Salmonella typhimurium* (strains TA97, TA98, TA100, TA102, and TA1535) compared to the vehicle control. Furthermore, EEZZR at doses of 150–5000 *μ*g mL^−1^ did not increase the number of structural aberrations in Chinese hamster lung cells in the presence or absence of S-9 mix. An oral administration of EEZZR to ICR mice, with doses of up to 2000 mg/kg, caused no significant increases in the number of micronucleated polychromatic erythrocytes (MNPCEs) and mean ratio of polychromatic erythrocytes to total erythrocytes. Lastly, RZZEE did not increase the incidence of MNPCEs in bone marrow. Based on these findings, it may be concluded that the use of EEZZR in traditional medicine poses no risk of genotoxicity.

## 1. Introduction

*Zingiber zerumbet* (L.) Smith (Zingiberaceae), commonly referred to as pinecone or shampoo ginger, is a perennial, tuberous root herb plant that can be found growing naturally in damp and shaded parts of the lowland or hill slopes, as scattered plants or thickets [1]. Despite its regular uses as a food flavoring and appetizer, *Z. zerumbet *rhizome (ZZR), in particular, has been used traditionally as a herbal medicine in Asian, Indian, Chinese, and Arabic folklores since ancient times [2]. Some traditional uses of RZZ include the treatment of inflammatory- and pain-mediated diseases, worm infestation, and diarrhea [3–5]. Furthermore, the methanol extract of ZZR possesses inhibitory effects on platelet-activating factor and Den2 virus NS2B/NS3 protease activity [6, 7]. A recent study has shown that the ethanol extract of ZZR (EEZZR) possesses antiobesogenic and antihyperlipidemic properties [8].

Despite the potential usefulness of herbal drugs, numerous reports on their adverse effects and fatalities have suggested that traditional herbal medicines need to be also evaluated for safety. Genotoxicity evaluations are one the most important nonclinical safety studies required for the registration and approval for marketing of pharmaceutical products. Furthermore, studies on the genotoxicity of medicinal plants that are used by the general population are warranted to identify the ingredients that pose mutagenic and carcinogenic risks. The active pharmacological component of *Z. zerumbet *rhizomes that is most widely studied is zerumbone [2]. It has been previously demonstrated that zerumbone is a cytotoxic, but not clastogenic, substance in cultured human peripheral blood lymphocytes [9]. Indeed, an acute and 28-day subchronic administration of EEZZR does not produce toxic effects in Wistar rats [10]. The genotoxic potential of RZZEE, however, has not been investigated thus far. In the present study, Ames, chromosomal aberration, and micronucleus tests were conducted to compare the safety of EEZZR and zerumbone.

## 2. Materials

### 2.1. Chemicals and Solutions

Zerumbone (purity  ≥98.0%) and 6-gingerol (purity  ≥98.0%) were purchased from Sigma-Aldrich Co. (St. Louis, MO, USA) for liquid chromatography-tandem mass spectrometry (LC/MS/MS) analysis. Most of the chemicals used for the genotoxicity evaluations of EEZZR, including 2-aminofluorene (2-AF), 2-aminoanthracene (2-AA), 9-aminoacridine hydrochloride (9-AA), sodium azide (SA), mitomycin C(MMC), benzo[a]pyrene (B[a]P), Giemsa, cyclophosphamide (CPA), and colcemid, were also purchased from Sigma-Aldrich Co. Eagle's minimum essential medium (MEM), fetal bovine serum (FBS), penicillin, and streptomycin were purchased from GIBCO-Invitrogen (Carlsbad, CA, USA). The rat liver microsomal enzyme, S-9, which was prepared from male Sprague-Dawley rat livers that were induced with Aroclor-1254, was obtained from Molecular Toxicology Inc. (Boone, NC, USA), and the cofactor for the S-9 mix was obtained from Wako Pure Chem. Ind., Ltd. (Japan). All chemicals were of analytical grade.

### 2.2. Plant Material and Extraction

The rhizomes of *Z. zerumbet* were purchased from a local market in Dongshan, Dongshan Dist. (Tainan City, Taiwan) in October 2010. Macroscopic and microscopic examinations, as well as thin-layer chromatography and high-performance liquid chromatography (HPLC), were used to confirm the authenticity of the plant material provided (performed by Dr. Hong, Department of Biotechnology, College of Pharmacy and Health Care, Tajen University). Random amplified polymorphic DNA analysis of the ZZR supplied was also performed to identify DNA polymorphisms. The voucher specimen (Lot no. ZZR20101018) was deposited in our laboratory. Extraction was performed by maceration and air-dried, and 5 kg of pulverized ZZR was added to 10 L of 95% ethanol at room temperature for 7 days, which was occasionally shaken. The ethanol extracts of ZZR (EEZZR) were evaporated to dryness under reduced pressure for the total elimination of alcohol, followed by lyophilization, yielding approximately 582 g of dry residue (w w^−1^ yield: 11.6%). EEZZR was kept at −20°C until use and diluted in distilled water. The samples were then analyzed using the LC/MS/MS protocol described below.

### 2.3. LC/MS/MS System

 Chromatographic separation was performed using an HPLC apparatus equipped with two Micropumps Series 200 (PerkinElmer, Shelton, CT, USA), a UV/VIS series 200 detector (PerkinElmer, Shellton, CT, USA) set at a wavelength of 280 nm, and a Prodigy ODS3 100A column (250 mm × 4.6 mm, particle size 5 *μ*m) (Phenomenex, CA, USA). The eluents were (a) 0.2% formic acid in water and (b) acetonitrile/methanol (60 : 40, v v^−1^). The following gradient program was used: 20–30% B (6 min), 30–40% B (10 min), 40–50% B (8 min), 50–90% B (8 min), 90-90% B (3 min), and 90–20% B (3 min) at a constant flow of 0.8 mL min^−1^. The LC flow was split, and 0.2 mL min^−1^ was sent to the mass spectrometer. Three 20 *μ*L injections were performed for each sample. MS and MS/MS analyses of EEZZR were performed on an API 4000 triple quadrupole mass spectrometer (Applied Biosystems, Canada), equipped with a TurboIonSpray source and functioning in the negative ion mode. Analyses were performed using the following settings: drying gas (air) set at 400°C, capillary voltage (IS) at 4000 V, nebulizer gas (air) at 12 (arbitrary units), curtain gas (N_2_) at 14 (arbitrary units), and collision gas (N_2_) at 4 (arbitrary units). To optimize the declustering potential, focus potential, and collision energy for each compound, standard solutions (10 *μ*g mL^−1^) were infused directly into the mass spectrometer at a constant flow rate of 5 *μ*L min^−1^ using a model 11 syringe pump (Harvard Apparatus, Holliston, MA, USA). Zerumbone or 6-gingerol at concentrations of 25 to 400 *μ*g mL^−1^ was used to construct the standard curve. The retention times of the main compounds were 8.48 and 7.52 min for zerumbone and 6-gingerol, respectively. The linearity of the peak area (*y*) versus concentration (*x*, *μ*g2009mL^−1^) curve for zerumbone or 6-gingerol was used to calculate the contents of the main components in EEZZR.

### 2.4. Bacterial Reverse Mutation Test (Ames Test)

The Ames test was conducted according to the methods described previously with minor modification [11, 12]. The histidine-dependent auxotrophic mutants of* Salmonella typhimurium* (strains TA97, TA98, TA100, TA102, and TA1535) used in this study were kindly supplied by Professor Jiunn-Wang Liao from the Graduate Institute of Veterinary Pathobiology, National Chung Hsing University (Taichung City, Taiwan). The assay was conducted by using the preincubation method in the presence and absence of S-9 metabolic activation with EEZZR at concentrations of 150–5000 *μ*g per plate, along with the negative (30% DMSO) and positive controls containing 2-AF (10 g per plate versus TA97 and TA102 with S-9), 2-AA (1 *μ*g per plate versus TA98, TA100, and TA1535 with S-9), 9-AA (0.2 *μ*g per plate versus TA97 without S-9), 2-AF (0.1 *μ*g per plate versus TA 98 without S-9), SA (1 *μ*g per plate versus TA100 and TA1535 without S-9), and MMC (0.5 *μ*g per plate versus TA102 without S-9). After incubating at 37°C for 48 h, the number of revertant colonies was determined. Test compounds were considered to be positive for mutagenicity if there was a twofold increase from the negative control value or a dose-dependent increase in the number of revertant in one or more strains.

### 2.5. Chromosomal Aberration Assay

 The chromosomal aberrations study was conducted according to the Organization for Economic Co-operation and Development (OECD) guidelines [13]. The experimental methods were based on previously published reports with minor modifications [14]. Chinese hamster lung (CHL) cells were obtained from Bioresource Collection and Research Center (BCRC 60183) of the Food Industry Research and Development Institute (Hsinchu, Taiwan). The cells were cultured in MEM supplemented with 2 mmoL L^−1^ of l-glutamine, 100 mg mL^−1^ of streptomycin, 10^5^ U of penicillin, and 10% FBS. Subcultures were conducted every 3-4 days to prevent overgrowth [14].

Cells were seeded at 1.0 × 10^5^ per plate after a 24 h incubation period the culture medium was replaced with fresh medium without FBS that contained the test substance dissolved in distilled water. For the short-term treatment, cells were exposed to each test substance for 6 h with either additional culture medium (without metabolic activation) or S-9 mix at a final concentration of 5% (with metabolic activation). After a 6 h exposure period, the cells were washed and then incubated in fresh medium for an additional 18 h. For the continuous treatment, cells were treated with each dose for 24 h or 48 h. Colcemid was added (at a final concentration of 0.1 *μ*g mL^−1^) 2 h prior to harvesting. The cells were then harvested, swollen in 75 mmoL L^−1^ of KCl solution for 20 min at 37°C. Cells were fixed and washed three times with acetic acid and ethanol (1 : 3) and then placed onto clean glass slides. Slides were stained with 3% of Giemsa, and 100 metaphases per slide (200 metaphases per dose) were analyzed. Chromosomal aberrations were morphologically identified according to the principles described in the Atlas of chromosome aberration by chemicals [15]. MMC and B[a]P were used as positive controls, and solvent-treated cells served as the negative control. After staining with trypan blue, the number of live cells was counted with a hemocytometer using a portion of the trypsin-treated solution used for the chromosome preparation. The cell growth index was calculated with the control being 100% and no live cells being 0%. Regardless of whether there was a presence of aberration, an additional 100 metaphases were examined to determine the frequency of polyploidy and endoreduplication.

### 2.6. Mouse Bone Marrow Micronucleus Assay

 The mouse bone marrow micronucleus assay was carried out as recommended by Schmid (1976) [16] and the OECD TG 474 Guidelines for Mammalian Erythrocyte Micronucleus Test [17] with minor modifications. Specific pathogen-free male and female ICR mice at 6 weeks of age (weighing 25.3–28.3 g) were obtained from the National Laboratory Animal Center (Taipei City, Taiwan) and used after one week of quarantine and acclimatization. They were maintained in a temperature-controlled room (25 ± 1°C) on a 12 h : 12 h light-dark cycle (lights on at 06:00 h) in the animal center (Tajen University, Pingtung County, Taiwan) and had free access to a standard commercial diet containing 60% vegetable starch, 5% fat, and 18% protein (Harlan Teklad; Cat. no. 2018), as well as tap water. All animal procedures were performed according to the Guidelines for the Care and Use of Laboratory Animals of the National Institutes of Health (United States), as well as the guidelines of the Animal Welfare Act. These studies were conducted with the approval of the Institutional Animal Care and Use Committee (IACUC) at Tajen University (approval number: IACUC 99-16; approval date: September 9, 2010).

The preliminary study showed that oral administration of EEZZR did not induce any toxic effects at a dose of 2000 mg kg^−1^. Based on these results, EEZZR was administered once a day for 2 days via gavage to ICR mice at doses of 500, 1000, and 2000 mg kg^−1^. Mice in the negative control group received only the vehicle (distilled water) by gavage. CPA in normal saline (10 mL kg^−1^) was administered via intraperitoneal injection at a dose of 70 mg kg^−1^ and served as a positive control [18, 19]. Each group contained six females and six males. All animals were observed daily for clinical signs, and the body weights of each animal were measured at the initiation of treatment and prior to bone marrow sampling. Animals were sacrificed by CO_2_ gas inhalation 24 h after the last administration. The bone marrow was expelled from the cavity by gentle aspirations and flushing with FBS using a disposable syringe with a 23G needle. The cell suspension was then centrifuged at 1000 rpm for 5 min, and the supernatant was discarded. After removing the supernatant, a small drop of the viscous suspension was smeared onto clean microscope slides. Preparations were air-dried and fixed by submerging in absolute methanol for 5 min. Fixed slides were stained with May-Grunwald and Giemsa. Stained slides were then rinsed with distilled water, dried, and mounted (Depex, Fluka). Slides were examined under 1000x magnification. Small round or oval bodies within the erythrocytes, with a size of about 1/5 to 1/20 of the diameter of a polychromatic erythrocyte (PCE), were regarded as micronuclei. The results were expressed as the number of micronucleated PCEs (MNPCEs; PCE with one or more micronuclei) per 2000 PCEs. Additionally, the PCE/(PCE + NCE) ratio, where NCEs denote normochromatic erythrocytes, was calculated by counting 500 cells.

### 2.7. Statistical Analysis

All data are expressed as mean ± standard deviation (SD) for the indicated number of experiments. Statistical analysis was not performed for bacterial reverse mutation and chromosome aberration assays. Statistical evaluation of the *in vivo* micronucleus results was performed according to a previous study [20] with minor modification. Differences in the number of MNPCEs between the treated and control groups were determined via the Kruskal-Wallis H-test and Dunn's Rank Sum test, where appropriate. The Mann-Whitney *U*-test was used to compare the PCE/(PCE + NCE) ratios of the treated and vehicle control groups. A *P* value <0.05 was considered statistically significant.

## 3. Results

### 3.1. Phytochemical Analysis

**Figure 1:**
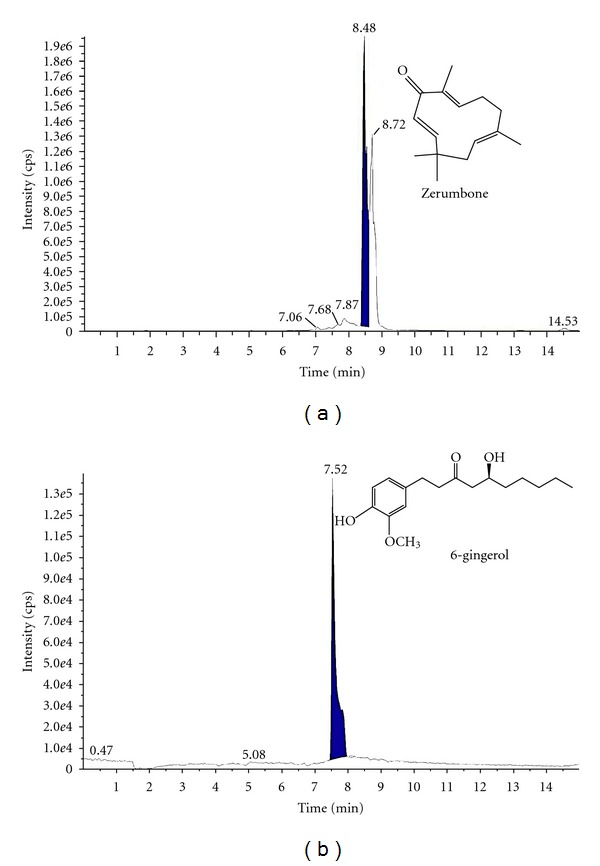
LC/MS/MS chromatogram for (a) zerumbone and (b) 6-gingerol in RZZEE sample.

The linear equations for zerumbone and 6-gingerol were *y*  =  52102*x* + 294730 (*R* ^2^ = 0.9908) and *y*  =  118041*x* + 271109 (*R* ^2^ = 0.9967), respectively. The chromatogram of the EEZZR solution is presented in Figure 1. The contents of zerumbone and 6-gingerol in EEZZR were 200.3 ± 0.37 and 102.5 ± 0.28 *μ*g g^−1^, respectively.

### 3.2. Bacterial Reverse Mutation Assay

The results of the Ames test are presented in Table 1. In all strains, the revertant numbers induced by EEZZR (150–5000 *μ*g per plate) were similar to those of the negative control, and none were greater than or equal to twofold of the negative controls. Therefore, these findings suggest that EEZZR is not mutagenic in several *Salmonella typhimurium* strains, specifically TA97, TA98, TA100, TA102, and TA1535.

### 3.3. Chromosomal Aberration Assay

The results of the chromosomal aberration assay are shown in Table 2. There were no significant changes in the structural and numerical chromosomal aberrations with any dose of EEZZR and treatment lasting 6, 24, and 48 h with or without S-9.

### 3.4. Mammalian Erythrocytes Micronucleus Test

In the micronucleus test using ICR mice, there were no abnormal signs in general appearance and body weights observed in mice between the first and final administration in the vehicle control, positive control, and 500, 1000, or 2000 mg kg^−1^ of EEZZR per day treatment groups (Table 3). Additionally, EEZZR did not induce any significant changes in MNPCEs, and there were no significant decreases in the PCE/(PCE + NCE) ratio at any dose of the EEZZRtreatment groups compared to the vehicle control.

## 4. Discussion

Traditional medicine is used to prevent, diagnose, improve, and treat illnesses. In general, folk medicine uses plant extracts without considering their potential toxicity. In our previous study, the 50% lethal dose of EEZZR was determined to be greater than 15 g kg^−1^ for a single oral dose in rats, and there were no adverse effects observed during a 4-week repeated oral dose toxicity study with a dose of 3000 g kg^−1^ [9]. In the present study, genotoxicity assessments were carried out using the bacterial reverse mutation (Ames test), chromosomal aberration, and mouse micronucleus tests to provide more safety information on EEZZR.

The Ames test is a biological assay used to assess the mutagenic potential of chemical compounds and complex environmental mixtures, and it is simple and quick in estimating carcinogenic potential. The potential of EEZZR in inducing reverse mutations in several* Salmonella typhimurium* strains (i.e., TA97, TA98, TA100, TA102, and TA1535) was evaluated by the Ames test. Alterations in the number of revertant colonies were not detected for concentrations of 150–5000 *μ*g per plate, regardless of the metabolic activation system used for each *Salmonella typhimurium* strain. However, the positive controls, specifically 2-AF, 2-AA, 9-AA, SA, and MMC, demonstrated significant mutagenicity with or without the metabolic activation system in some of the strains. Thus, our results indicate that EEZZR does not induce mutagenicity in several strains of *Salmonella typhimurium*, as determined by the Ames test.

Then, a chromosomal aberration test using cultured CHL cells was performed with or without a metabolic activation system. There were no significant differences in the incidence of chromosomal aberrations, even after a 48 h treatment with the highest concentration of EEZZR at 5000 *μ*g mL^−1^ without the S-9 mix. However, the positive control groups demonstrated significant increases in the frequency of metaphases with aberrant chromosomes. Therefore, EEZZR did not induce any changes in the chromosome aberration assay under the conditions of this study.

Bone marrow is the site of rapid cell proliferation, and hence it is the most preferred organ for genotoxicity evaluations of different chemicals [21]. In mice, micronuclei are evident in circulating red blood cells, unlike in rats and humans, as their spleens do not remove red blood cells containing micronuclei [22]. There were no differences in the incidence of micronuclei of mice treated with this extract at 500, 1000, and 200 mg kg^−1^ per day, as determined by the micronucleus assay. However, the positive control group demonstrated significantly higher levels of micronuclei compared to those of the negative control. From these results, it can be concluded that EEZZR does not possess mutagenic potential in the micronucleus assay. Thus, the results of the *in vivo* assay corroborate those of the *in vitro *mutagenicity test. Both assays strongly suggest that the consumption of EEZZR does not pose any genotoxic hazards.

The rhizomes of *Z. zerumbet* have been the subject of extensive chemical investigations due to their high medicinal values. The chemical constituents that are more frequently found in EEZZR are flavonoids, such as kaempferol, quercetin, and curcumin [23]. There are numerous reports in the literature on the safety of phenolics found in medicinal plants, which contain beneficial health effects [24]. However, studies on the genotoxicity of flavonoids suggest that they possess both protector and inductor genotoxic effects depending on the specific compound and/or assay used. For instance, quercetin has been found to be genotoxic in TA98 and TA100, but it was also shown to reduce the clastogenicity effect of B[a]P in mice [25, 26]. Our results reveal that EEZZR, which contains phenolic compounds, did not cause any genotoxic effects, as determined by the Ames test, *in vitro* chromosomal aberration assay, and *in vivo* micronucleus assay. Indeed, zerumbone is the main common component in leaves and rhizomes oils, and a considerable amount of 6-gingerol was also found in EEZZR. Genotoxic activity has not yet been associated with zerumbone and 6-gingerol. However, some reports regarding their chemopreventive and antimutagenic effects have been previously published [27–29]. It is important to note that the positive *in vitro *mutagenicity tests are not necessarily positive *in vivo*, as many substances with mutagenic activity cannot be absorbed, and thereby their mutagenic potential is minimized [30].

The present study suggests that EEZZR is not mutagenic in the *in vitro Salmonella*/microsome assay, does not induced chromosomal aberrations in CHL cells, and does not induce and increase the incidence of micronucleated polychromatic erythrocytes in mouse bone marrow. Additionally, there were no dose-dependent effects observed in any of the parameters measured, suggesting that EEZZR does not possess treatment-related adverse effects. Our findings support the notion that EEZZR is safe with respect to genotoxicity and general toxicity, if individuals are provided with the proper dose of EEZZR.

## Figures and Tables

**Table 1:** Results of bacterial reverse mutation assay.

Treatments	Dose (*μ*g per plate)	Revertant colonies per plate				
		TA97	TA98	TA100	TA102	TA1535
EEZZR without S9	0	156 ± 12	20 ± 3	105 ± 10	274 ± 12	13 ± 3
	150	167 ± 14	22 ± 2	110 ± 8	282 ± 14	15 ± 4
	300	166 ± 10	21 ± 3	105 ± 11	276 ± 18	14 ± 3
	600	173 ± 18	20 ± 5	104 ± 9	268 ± 17	12 ± 5
	1250	176 ± 11	22 ± 2	112 ± 12	276 ± 10	14 ± 6
	2500	169 ± 16	23 ± 4	108 ± 15	258 ± 13	14 ± 8
	5000	168 ± 11	21 ± 7	103 ± 16	261 ± 11	13 ± 6

EEZZR with S9	0	177 ± 16	21 ± 4	105 ± 12	282 ± 14	15 ± 4
	150	183 ± 14	23 ± 3	110 ± 11	290 ± 18	16 ± 5
	300	188 ± 17	22 ± 6	105 ± 15	284 ± 17	15 ± 7
	600	186 ± 12	21 ± 4	104 ± 12	276 ± 12	14 ± 6
	1250	197 ± 15	23 ± 6	112 ± 14	287 ± 13	17 ± 8
	2500	182 ± 14	24 ± 7	108 ± 16	265 ± 15	16 ± 9
	5000	191 ± 16	22 ± 6	103 ± 13	268 ± 16	16 ± 4

Positive controls						
2-AF with S9	10	1260 ± 48			966 ± 36	
2-AA with S9	1		697 ± 21	847 ± 29		127 ± 18
9-AA without S9	0.2	1096 ± 32				
2-AF without S9	0.1		993 ± 28			
SA without S9	0.5			774 ± 33		508 ± 27
MMC without S9	0.5				1640 ± 52	

Values are mean ± SD of 3 plates.

**Table 2:** Results of the chromosomal aberration assay in CHL cells.

Exposure (h)/±S9	Dose (*μ*g mL^−1^)	Structural aberrations		Numerical aberrations		Cell growth (%)
		Chromatid break	Chromatid exchange	Polyploidy	Endoreduplication
6/+S9	0	1	0	1	0	100
	150	2	0	1	0	99.2
	300	2	0	1	0	98.3
	600	1	0	0	0	99.4
	1250	2	0	1	0	98.5
	2500	1	0	0	0	99.1
	5000	3	1	1	0	97.8
	B[a]P 0.2	25	37	6	2	61.3

6/−S9	0	1	0	0	0	100
	150	3	0	2	0	99.3
	300	3	0	1	0	98.2
	600	2	0	1	0	97.6
	1250	2	0	1	0	98.1
	2500	3	0	0	0	97.4
	5000	3	1	0	0	96.5
	B[a]P 0.15	25	36	5	2	57.2

24/−S9	0	0	0	1	0	100
	150	4	0	1	0	99.3
	300	3	0	2	0	97.9
	600	1	0	0	0	98.2
	1250	2	0	1	0	96.8
	2500	1	0	0	0	97.4
	5000	3	0	2	0	98.6
	MMC 0.05	36	44	3	1	62.3

48/−S9	0	0	0	1		100
	150	0	0	0	0	99.6
	300	1	0	1	0	98.2
	600	3	0	1	0	98.1
	1250	3	0	0	0	97.9
	2500	1	0	0	0	98.2
	5000	1	0	1	0	97.6
	MMC 0.05	26	0	4	1	60.4

**Table 3:** Results of micronucleus test.

Treatments	Dose (mg kg^−1^)	MNPCE/2000 PCEs	PCE/(PCE + NCE)
Vehicle	0	1.66 ± 1.05	0.52 ± 0.03
EEZZR	500	1.65 ± 1.13	0.54 ± 0.05
	1000	1.58 ± 0.98	0.52 ± 0.04
	2000	1.60 ± 1.11	0.51 ± 0.06
CPA	70	62.31 ± 5.02*	0.42 ± 0.09

Data are presented as mean ± SD from twelve mice (6 male mice and 6 female mice) per dose group. Since there was no difference between males and females within the same dose group, Table 3 shows the combined data for males and females. ^∗^Significantly different from the control at *P* < 0.05. Bone marrow cells were harvested 24 h after the last oral dose of EEZZR or CPA injection.
